# Effects of Genetically Modified Milk Containing Human Beta-Defensin-3 on Gastrointestinal Health of Mice

**DOI:** 10.1371/journal.pone.0159700

**Published:** 2016-07-20

**Authors:** Xin Chen, Yange Yang, Zhaopeng Shi, Ming-Qing Gao, Yong Zhang

**Affiliations:** 1 College of Veterinary Medicine, Northwest A&F University, Yangling 712100, Shaanxi, China; 2 Key Laboratory of Animal Biotechnology, Ministry of Agriculture, Northwest A&F University, Yangling 712100, Shaanxi, China; Kurume University School of Medicine, JAPAN

## Abstract

This study was performed to investigate the effects of genetically modified (GM) milk containing human beta-defensin-3 (HBD3) on mice by a 90-day feeding study. The examined parameters included the digestibility of GM milk, general physical examination, gastric emptying function, intestinal permeability, intestinal microflora composition of mice, and the possibility of horizontal gene transfer (HGT). The emphasis was placed on the effects on gastrointestinal (GI) tract due to the fact that GI tract was the first site contacting with food and played crucial roles in metabolic reactions, nutrition absorption and immunity regulation in the host. However, the traditional methods for analyzing the potential toxicological risk of GM product pay little attention on GI health. In this study, the results showed GM milk was easy to be digested in simulated gastric fluid, and it did not have adverse effects on general and GI health compared to conventional milk. And there is little possibility of HGT. This study may enrich the safety assessment of GM product on GI health.

## Introduction

Transgenic technology has been applied in medicine, research, industry and agriculture and can be used on a wide range of plants, animals and micro organisms. In animal science, gene manipulation is mainly applied in the aspects of breeding for disease resistance [1, 2], improving product performance [3, 4], and producing medical protein [5] by modifying the genetic materials of the target animals which are referred as to genetically modified (GM) animals. The application and promotion of transgenic products require both governmental approval and public understanding and support. The decision about GM food is safe has been suspended although the GM products have been existed from many years. For instance, the concern may arise whether the GM products have equal nutrients to the conventional products and equal effects on consumer health [6]. This is greatly due to that when the genome of animal is modified to obtain the intended traits, additional traits could be acquired and existing traits may be lost or modified. All these changes may be neutral, beneficial, or deleterious with respect to the health of the animal and the safety of the products which derived from the animal. Recently, several studies reported that exogenous gene of the GM products may transfer to intestinal microflora and further possibly enter into the environment [7–9], which was also found in conventional breeding animals [10]. Therefore, GM products should undergo a regulatory of safety assessment before they are allowed into the market [6].

The safety assessments of GM products generally include toxicity studies [11, 12], nutrient composition analysis [13, 14], and protein allergenicity analysis [15]. Traditionally, the toxicity study of GM products is assessed by an acute toxicity test of a 30-day feeding study or a subchronic toxicity test of a 90-day feeding study on animals that mainly focus on the physiological indexes of the experimental animals, such as body weight, food utilization, blood and urine chemistry, hematology, organ weight, and gross necropsy [11, 16, 17]. However, these indexes are with less focus on the specific physiology and pathophysiology of the gastrointestinal (GI) tract which is the first site for contacting with the outer environment, food and bacteria [18]. The GI tract has a large amount of intestinal microflora colonized the surface of enterocytes which participate in food digestion and absorption, and form the immunoprotective biological barrier [19]. The balance of microflora in GI tract is crucial for host health maintenance, and the perturbation of the microbial composition has been hypothesized to be involved in a range of diseases [20]. Therefore, it would be truly essential for GM product safety assessments to take account the effects of GM products on GI tract and the microflora composition of the intestine.

Human beta-defensin-3 (HBD3), a kind of antimicrobial peptide, has broad spectrum antimicrobial activity against bacteria, fungi, and enveloped viruses, and has an important role in immunity [21]. HBD3 shows antibacterial activity against both *Staphylococcus aureus* (*S*. *aureus)* and *Escherichia coli* (*E*. *coli)* at very low concentrations [22]. *S*. *aureus and E*. *coli* are two major mastitis causing pathogens, and bovine beta-defensin-3 is not normally expressed in cow’s milk. Thus, a nonhuman source of HBD3 could provide important applications due to these antimicrobial activities.

Mastitis is the most common productive disease of cow. It causes great economic losses due to decreases in the quality and quantity of milk production, increases in the cost of treatment. The common treatment of mastitis is antibiotics. However, the various pathogenic bacteria species and infection mechanism make the treatment effect not very ideal. Using high doses of antibiotics for a long time will also cause many problems such as more drug-resistant strains and antibiotic residues.

Our research group has produced twelve healthy transgenic cattle expressing HBD3 using somatic cell nuclear transfer via a site-specific recombinase-based method [23], and these cattle showed enhanced resistance to the infection of bacteria by expressing HBD3 in mammary gland. And the ability of transgenic cow to resist mastitis was tested by intramammary infusion of vibale bacterial cultures. The transgenic cow with the highest HBD3 expression level never became infected after bacteria infusion, while 27 of 30 glands of conventional cow became infected. The milk produced by these transgenic dairy cows contains HBD3, and the milk repressed the growth of both *S*. *aureus* and *E*.*coli*. As the milk ingredients were changed, we need to evaluate whether GM milk was as safe as conventional milk to health before the GM milk was permitted to enter into the market. In the present study, a 90-day feeding study in C57BL/6 mice on the test material prepared using GM milk containing HBD3 were carried out to study the effects of the GM milk on mice, and we just focused on five transgenic cattle that were lactating naturally during the experimental period. Apart from the physiological indexes of the mice such as body weight, food consumption, blood chemistry, and organ weight, the emphasis was placed on the effects of HBD3 milk on the physiological and pathophysiological condition of the GI health and the intestinal microbial composition.

## Materials and Methods

### Milk samples

Both conventional milk and GM milk were acquired from Experimental Base of Northwest A&F University (Yangling Keyuan Cloning Co., Ltd., China). Notably, the GM milk was not commercial and only for scientific research before it was permitted to enter into the market. The concentration range of HBD3 in GM milk of five transgenic cows was from 3.9 to 10.4 μg/ml and no significant decline of HBD3 was observed during the natural lactation period of 6 months [23]. The GM milk with highest concentration of HBD3 was used in this study. The conventional milk was used as control. These two types of milk was pasteurized at 73.8°C, and then immediately cooled and stored at 4°C for no more than a week before use.

### Experimental animals and diets

One hundred male and 100 female C57BL/6 mice (SPF) were obtained from Laboratory Animal Center of Fourth Military Medical University (Xi’an, Shaanxi, China). All mice were four weeks old at the start of treatment, and were housed in a polypropylene plastic cage with *ad libitum* access to water and food. The room was maintained a constant environment with temperature at 22 ± 2°C, relative humidity of 50 ± 10%, air change 5 times/h, and a 12 h light/dark cycle. The mice were divided into control group, GM groups, and non-GM group. The mice in control group consumed only the AIN93G diet that were prepared in our laboratory according to the standard protocol. The mice in GM groups were fed diets with addition of 10% and 30% (w/w) GM milk according to the AIN93G diet, while those in non-GM group were fed diets containing the same concentrations of conventional milk. For convenience to description, the groups fed AIN93G diet, AIN93G diet with 10% HBD3 milk addition, AIN93G diet with 30% HBD3 milk addition, AIN93G diet with 10% conventional milk addition, and AIN93G diet with 30% conventional milk addition was designated as C, 10G, 30G, 10N, and 30N group, respectively.

The protocol for the study was approved by the Animal Care Commission of the college of Veterinary Medicine, Northwest A&F University. All the animals obtained human care, and the study was executed in accordance with the institution’s guidelines.

### *In vitro* digestion of HBD3 milk

HBD3 milk was skimmed and used for *in vitro* analysis according the previously described protocol with slight modification [14, 15, 24]. Simulated gastric fluid was prepared by dissolving human pepsin (800–2500 U/mg, Sigma, St. Louis, MO, USA) into 0.03 mol/L NaCl solution and adjusted pH value to 2 with HCl, and the final concentration of pepsin is 500 mg/ml. Every 500 μl skim milk were mixed with 10 μl simulated gastric fluid, and incubated in a water bath at 37°C for 0, 1, 5, 15, 60, 120 min, respectively. The enzymatic reactions were stopped by adjustment of pH to 7 with 1 mol/L NaOH. The digestion products at 0, 1, 5, 15, 60, 120 min were separated by 10% Tricine-SDS-PAGE. Proteins were visualized by Coomassie Brilliant Blue staining. In western blot analysis, a rabbit polyclonal antibody against HBD3 (1:1000 dilution, Sigma, St. Louis, MO) was used as primary antibody and horseradish peroxidase-conjugated goat anti-rabbit IgG as the second antibody (1:1000 dilution, Beyotime, Jiangsu, China).

### General health evaluation

During the period of the feeding experiment, every mouse was observed daily for the signs of morbidity or clinical signs of toxicity. Body weight and food consumption of each mouse were measured weekly. At the end of examination, mice were anesthetized and killed by cervical dislocation. After a throughout necropsy, the main organs were excised, weighed. Serum biochemistry parameters were measured with an auto-analyzer (Hitachi 7180, Tokyo, Japan). Before collecting the blood samples, mice were fasted overnight with water *ad libitum*. The mice were anesthetized and blood samples were collected from hearts.

### Gastric emptying assay

The gastric emptying assay was performed as described previously [25]. Before the gastric emptying assay, mice were fasted for 14 h with water free to access. Two hundred microliter standard liquid test meal containing a nonabsorbable marker (0.5 mg/ml phenol red in 5% glucose solution) was poured into the stomach by a feeding needle. Mice were killed by cervical dislocation after 30 min of the gavage. Stomachs and small intestines were exposed and removed, and each small intestine was divided into four equal-length segments. The absorbance of phenol red was read at wavelength of 560 nm and the gastric emptying rate was calculated according to the formula: the amount of phenol red recovered in stomach/ total amount of phenol red recovered from stomach and small intestine.

### Transmission electron microscopy

All intestinal segments were cut into two-millimeter samples and then fixed in 4% buffered glutaraldehyde for 2 h. Following the fixation, the samples were post-fixed in 1% osmium tetroxide, sequentially dehydrated through an ascending alcohol series, and infiltrated in epoxy (SPI-Chem/ SPI-PON 812 KIT, West Chester, PA). The samples were embedded in resin at 30°C for 24 h and then 60°C for 48 h. Ultrathin sections were cut and stained by uranyl and lead citrate. The sections were examined and photographed on Hitachi HT7700 (Tokyo, Japan).

### Real-time quantitative PCR analysis of zo-1, occludin and claudin-1

Total RNA was extracted from each sample using TRIZOL reagent (Invitrogen, Carlsbad, CA), and reverse transcription was performed to generate cDNA using a PrimeScript^™^ RT Reagent Kit (TaKaRa, Dalian, China). Real-time PCR was performed using SYBR Premix Ex Taq^™^ (TaKaRa) as previous description [23]. Primer sequences targeting the zonula occluden 1 (zo-1), occludin, claudin-1 gene and house-keeping gene of GAPDH were listed in S4 Table. The amount of target normalized to the reference was calculated by the 2^-ΔΔCt^ method [23].

### Immunofluorescence

Immunofluorescence staining of intestinal tissues was performed according to the method described previously [26]. Intestinal tissues were washed with cold PBS, fixed in 3% paraformaldehyde overnight, embedded in paraffin, and cut into 5 μm thick serial sections. The sections were blocked with 5% normal goat serum in PBS containing 0.05% Tween-20 and 0.1% bovine serum albumin for 30 min, and then incubated with polyclonal goat anti-occludin (1:100 dilution, Santa Cruz Biotechnology, Dallas, TX), polyclonal goat anti-zo-1 (1:100 dilution, Santa Cruz Biotechnology) or polyclonal mouse anti-claudin-1 (1:100 dilution, Santa Cruz Biotechnology) in PBS with 1% goat serum overnight at 4°C. After washing three times by PBS, sections were incubated with Alexa 555-conjugated donkey anti-mouse IgG or Cy3-conjugated donkey anti-goat IgG for 60 min. DAPI was used for staining cell DNA.

### Tracer experiments

EZ-link Sulfo-NHS-Biotin (Pierce Chemical, Rockford, IL) was specific to label the cell surface proteins, and it was not able to permeate the intact cell membrane. The working concentration of biotin was 2 mg/ml in PBS containing 1mmol/L CaCl_2_. We ligated 1 cm middle part of ileum and colon after the animals were sacrificed. Biotin was slowly injected into the ligated parts and incubated for 5 min. Then the ligated parts were excised and placed into 4% paraformaldehyde in PBS (pH 7.3) for 3 h. After washing three times, 5 μm frozen tissue sections were made by freezing microtome (CM 1900, Leica Microsystems, Heidelberg GmbH, Mannheim, Germany). Finally, sections were incubated with streptavidin conjugated to Alexa 488 for 30 min [27].

### Denaturing gradient gel electrophoresis analysis of intestinal microflora profile

The variable V3 region of bacterial 16S rDNA was amplified with PCR as described previously [7] using GC-338F and 518R primer pair and the microbial DNAs template which were extracted from intestinal contents of randomly selected mice without regard to gender (5 mice/group). Denaturing gradient gel electrophoresis (DGGE) analysis was performed as reported before [7, 28]. Sequences of DGGE band were analyzed in the GenBank database using BLAST (www.ncbi.nlm.nih.gov/BLAST). DGGE bands were analyzed by Quantity One analysis software to calculate the preliminary data. Shannon diversity index was used to assess the diversity of the microflora. Clustering analysis was performed using the unweighted pair group method with arithmetic mean clustering algorithm (UPGMA).

### Horizontal gene transfer detection

DNA from intestinal bacteria and tissues of heart, liver, spleen, lung, kidney, muscle and intestine were used in horizontal gene transfer (HGT) detection. The mouse beta-actin gene was chosen as the reference gene. GC-338F and 518R were chosen as the bacteria universal primers. HBD3 gene and another fragment HBD3-CSN from the vector of transgenic cattle were treated as the target of the detection. PCR procedure and primers were in S5 Table, and reaction volumes were described in S6 Table.

### Statistical analysis

All data were analyzed by one-way ANOVA and least-significant difference tests using SPSS 20.0 statistical software (IBM Corporation, Somers, NY). All the results were presented as the mean ± SD. P < 0.05 was considered as statistically significant different.

## Results

### GM milk was unstable under the digestion of simulated gastric fluid *in vitro*

**Fig 1:**
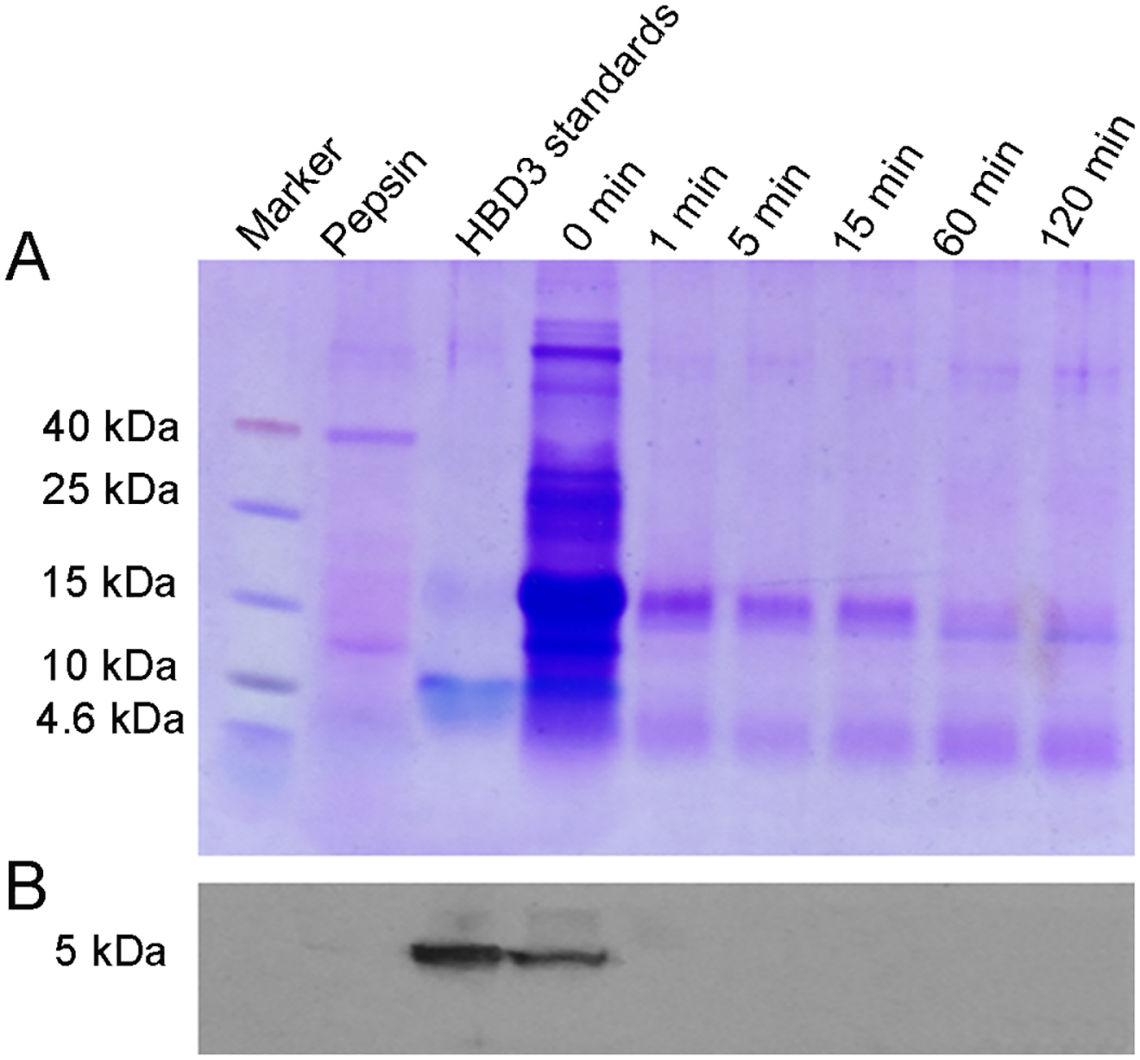
GM milk was digested by simulated gastric fluid *in vitro*. (A) Tricine-SDS-PAGE analysis and (B) Western blot analysis of the degradation of GM milk containing HBD3 in simulated gastric fluid. The blot was probed with polyclonal antibody to HBD3. Pepsin was 35 kDa, and HBD3 standards was 5 kDa. (Notably, the standard protein marker indicated by the location in gel was bigger than its real molecular weight about 5 kDa.)

Digestion stability has been proposed as one of the major criteria of the safety assessment of GM food [15, 29]. To test whether HBD3 affected the digestibility of GM milk, an *in vitro* digestion assay was established by using human pepsin as simulated gastric fluid. The products digested by simulated gastric fluid was separated by Tricine-SDS-PAGE gel and showed that no band was observed at 5.0 kb position at all checked time point based on the HBD3 standard, while an evident HBD3 band was represent at 0 min, indicating that HBD3 in GM milk was unstable under the digestion of simulated gastric fluid in vitro and was able to be completely digested with as less as 1 min (Fig 1A), and this finding was further convinced by western blotting (Fig 1B).

### GM milk has no adverse effects on general health conditions of mice

**Fig 2:**
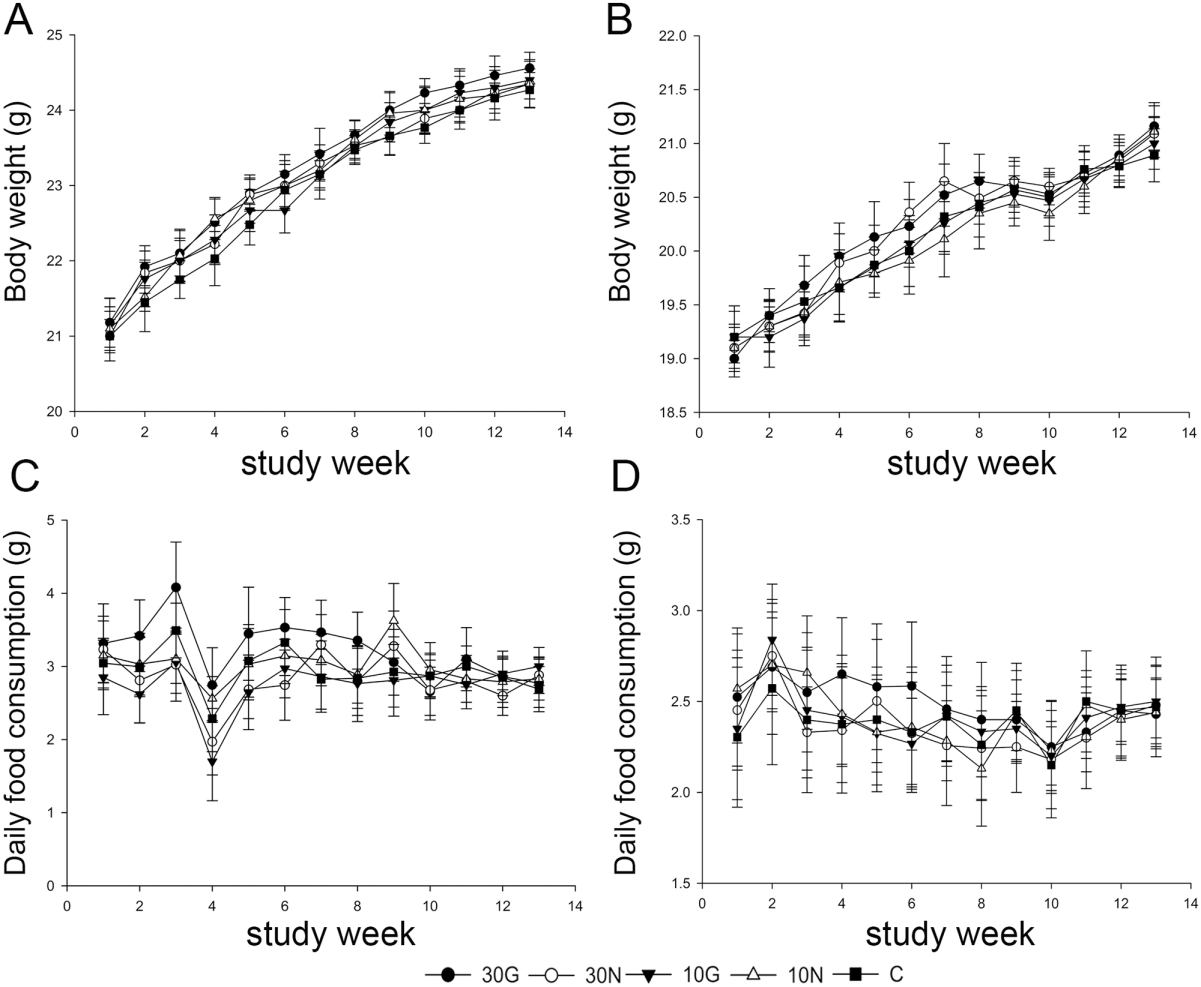
Body weight and daily food consumption. (A) Mean body weight of male mice (n = 20); (B) Mean body weight of female mice (n = 20); (C) Mean daily food consumption of male mice (n = 20); (D) Mean daily food consumption of female mice (n = 20).

During the course of the study, no clinical signs of adverse or toxic effects were observed in all groups. Experienced anatomical pathologist did not find macroscopic pathological changes through the necropsy, and no remarkable gross lesions were found in any organ. Data of the body weight and food consumption recorded weekly throughout the study period showed that both body weight and the food consumption were comparable among all groups (Fig 2). Relative organ weight was presented as the percentage of specific organ weight to the individual body weight, and there was no statistically significant difference in relative organ weight with an exception that the relative spleen weight of male mice in 30G group was higher than C group (P < 0.05) (S1 Table).

Serum biochemistry, which reflects the health condition (e.g., metabolism and physiological function) of the experimental animals, was also monitored. The result showed that no significant difference was observed in the most examined parameters of serum biochemistry except for triglyceride (TG) (S2 and S3 Tables). TG levels of mice in 30G group, no matter male or female, was higher than those in the C group. We should note that TG level of male mice in 30N group was also higher than those in C group.

### Gastric emptying function of mice fed with GM milk was normal

**Fig 3:**
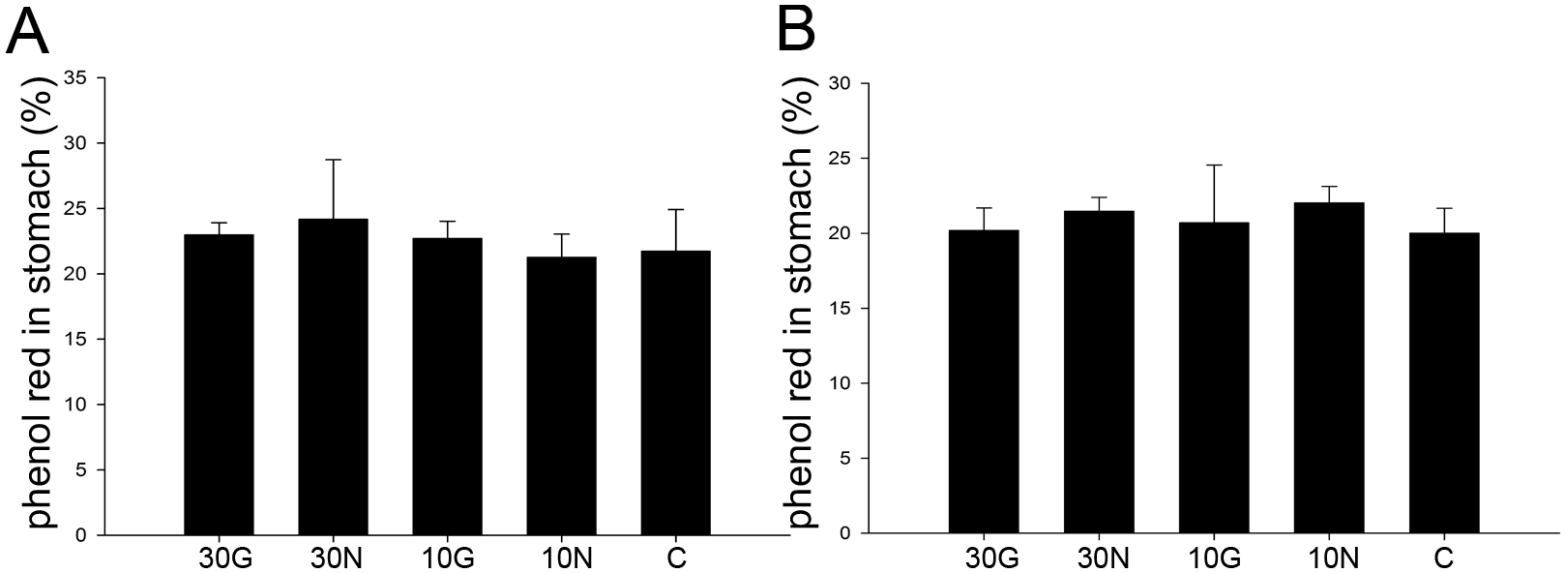
Gastric emptying function of mice *in vivo*. Percentage of phenol red in stomach in gastric emptying assay of male (A) and female (B) mice. The result was showed as the rate of the amount of phenol red in stomach/ the total amount of phenol red in stomach and small intestine.

The normal motor functions of the stomach include reservoir, emptying of liquids, solids and indigestible remnants, grinding, and liquefaction [30]. To evaluate the GI transit *in vivo*, we studied the dye of phenol red movement which is attributable to peristaltic propulsion by monitoring the gastric emptying rate. The result showed that all the groups exhibited identical gastric emptying ability (Fig 3), indicating that different diets had no effect on the gastric emptying function.

### Tight junctions of intestinal epithelium were intact

**Fig 4:**
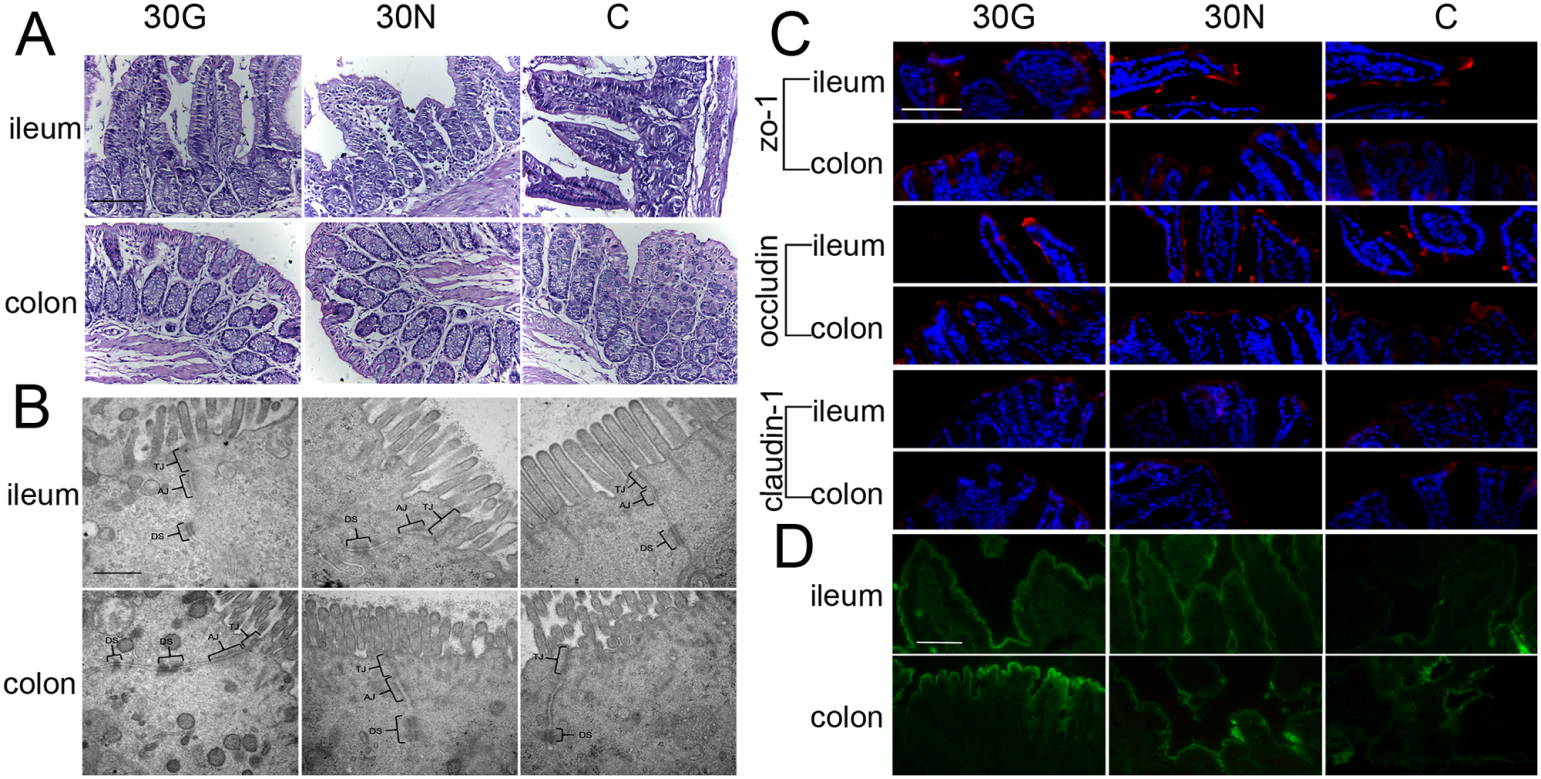
The analysis of intestinal permeability in ileum and colon of male mice. (A) Histopathological results of HE staining. Scale bar = 50 μm. (B) The ultrastructure were examined by transmission electron microscope. TJ: tight junctions. AJ: adherens junctions. DS: desmosomes. Scale bar = 400 nm. (C) Result of immunofluorescence of zo-1, occludin, and claudin-1 in intestinal epithelial cells. Frozen sections of ileum and colon were labeled for zo-1 (red), occludin (red), claudin-1 (red) and nuclei (blue). Scale bar = 100 μm. (D) Tracer experiment to examine the permeability in ileum and colon. The green fluorescence signals of biotin were found to be restricted to the lumen of the ileum and colon in each group. Scale bar = 50 μm.

Intercellular tight junction (TJ) structure located at the apical ends of the lateral membranes of the intestinal epithelial cells is the most important part of the selective permeable barrier of GI epithelium, and TJs are formed by multiple protein complexes including occludin, claudin-1 and zo-1 [31]. In this study, the permeability of the intestine was examined under light microscopy and transmission electron microscope. The images of HE staining under light microscopy showed that no histopathological change occurred in duodenum, jejunum, ileum, cecum and colon of mice in each group. Representative images were showed in Fig 4A, and the all images of HE staining of duodenum, jejunum, ileum, cecum and colon of male and female mice were provided as S1 Fig. The ultrastructure of intestine under transmission electron microscope showed that almost all epithelial cells presented typical intact TJ structures presented as a series of focal contacts between the plasma membranes of adjacent cells. In addition, adherens junctions and desmosomes were intact without structure destruction. TJ structures occasionally were discontinuous with few membrane fusions in each group. Representative images were showed in Fig 4B, and the all images of ultrastructure of duodenum, jejunum, ileum, cecum and colon of male and female mice were showed in S2 Fig. Furthermore, the mRNA expression levels of three constitutive proteins of TJ, zo-1, occludin and claudin-1, were analyzed by real time PCR. The result showed that mRNA expression level of each gene was identical in all segments of the intestine (S3 Fig). To further confirm the integrity of TJ structure, immunofluorescence study was carried out to detect the distribution of zo-1, occludin, and claudin-1 in the ileum and colon from mice after treatment with high concentration diets (30G and 30N) and control groups. The images of immunofluorescence staining showed that these three proteins were presented at the surface of epithelial cells to form a continuous barrier, and rarely found in cytoplasm, and the distributions of each protein were indistinguishable in all groups, indicating different diets did not cause abnormal expression and redistribution of zo-1, occludin and claudin-1 in ileum and colon. Representative images were showed in Fig 4C, and the all images of immunofluorescence of zo-1, occludin, and claudin-1 in ileum and colon of male and female mice were showed in S4 Fig. Moreover, we performed biotin-tracer experiments to visually assess the permeability of the ileum and colon epithelium. Tracer molecule biotin was poured into lumen and labeled with Alexa 488-conjuncted streptavidin. The biotin fluorescent signals were found to be restricted to the lumen of the ileum and colon in all groups. Representative images were showed in Fig 4D, and the all images of tracer experiment of ileum and colon of male and female mice were showed in S5 Fig. Taken together, these results indicated that GM milk did not affect the permeability of the intestine.

### The microflora diversity showed comparable differences between GM groups and Non-GM groups

**Fig 5:**
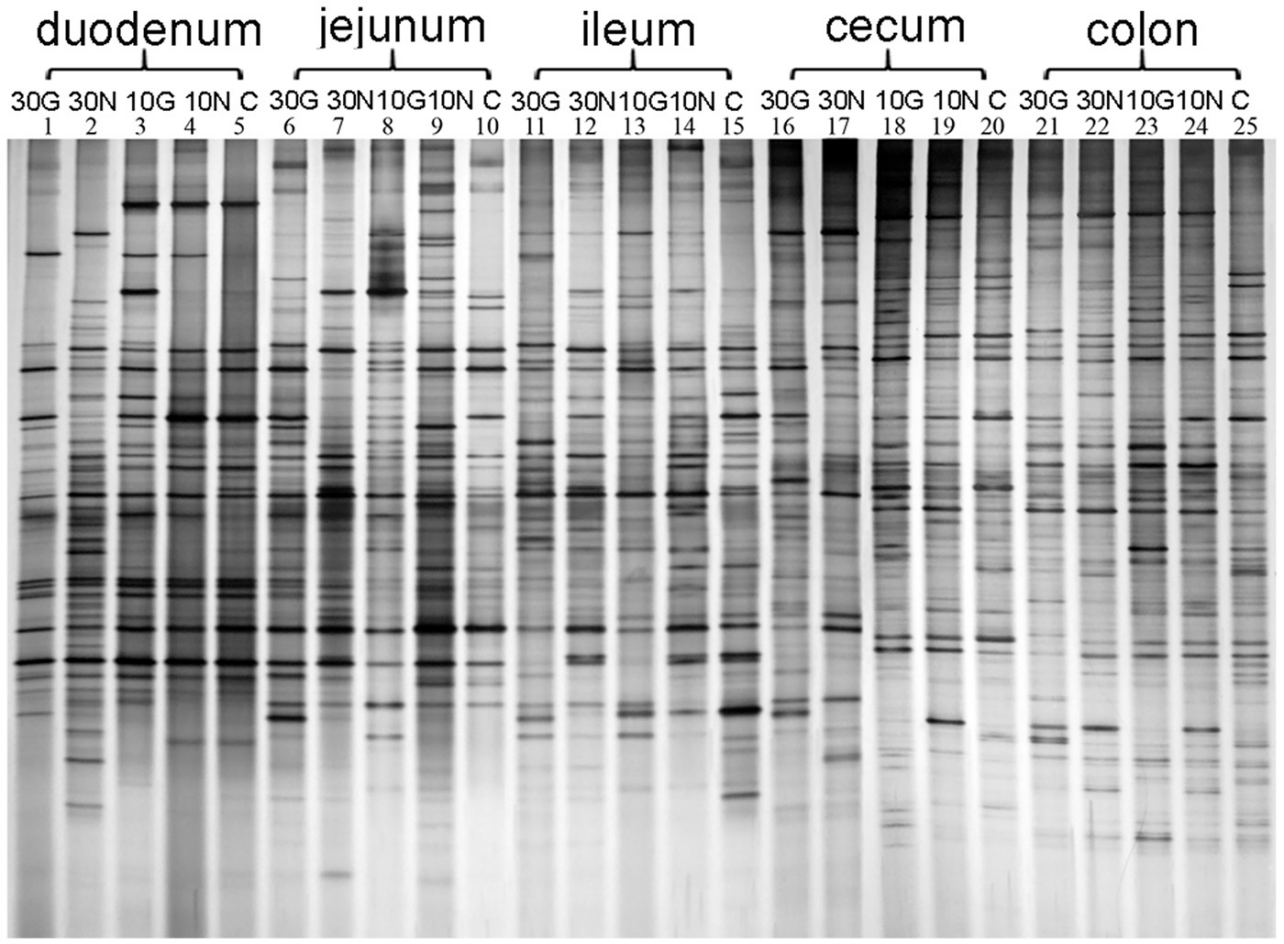
DGGE electrophoretogram of 16S rDNA PCR products from intestinal content samples. The numbers 1 to 25 on the top of the electrophoretogram indicated as following: the 1–5 represented the samples of duodenum of 30G, 30N, 10G, 10N, and C groups. 6–10 represented the samples of jejunum of 30G, 30N, 10G, 10N, and C groups. 11–15 represented the samples of ileum of 30G, 30N, 10G, 10N, and C groups. 16–20 represented the samples of cecum of 30G, 30N, 10G, 10N, and C groups. 21–25 represented the samples of colon of 30G, 30N, 10G, 10N, and C groups. Every sample was collected from 5 mice to treat as a sample pool.

The large and dynamic bacterial community which resides in GI tract, also known as microflora, has great effect and relevance on physiology and pathology of the host [32]. At the termination of experiment (day 90), the mice were under necropsy and the contents of the duodenum, jejunum, ileum, cecum, and colon from all groups without regarding to gender were analyzed by DGGE. Five individual samples from each group were mixed and treated as a sample pool, and each sample pool has a unique microflora composition with a different band profile (Fig 5).

**Fig 6:**
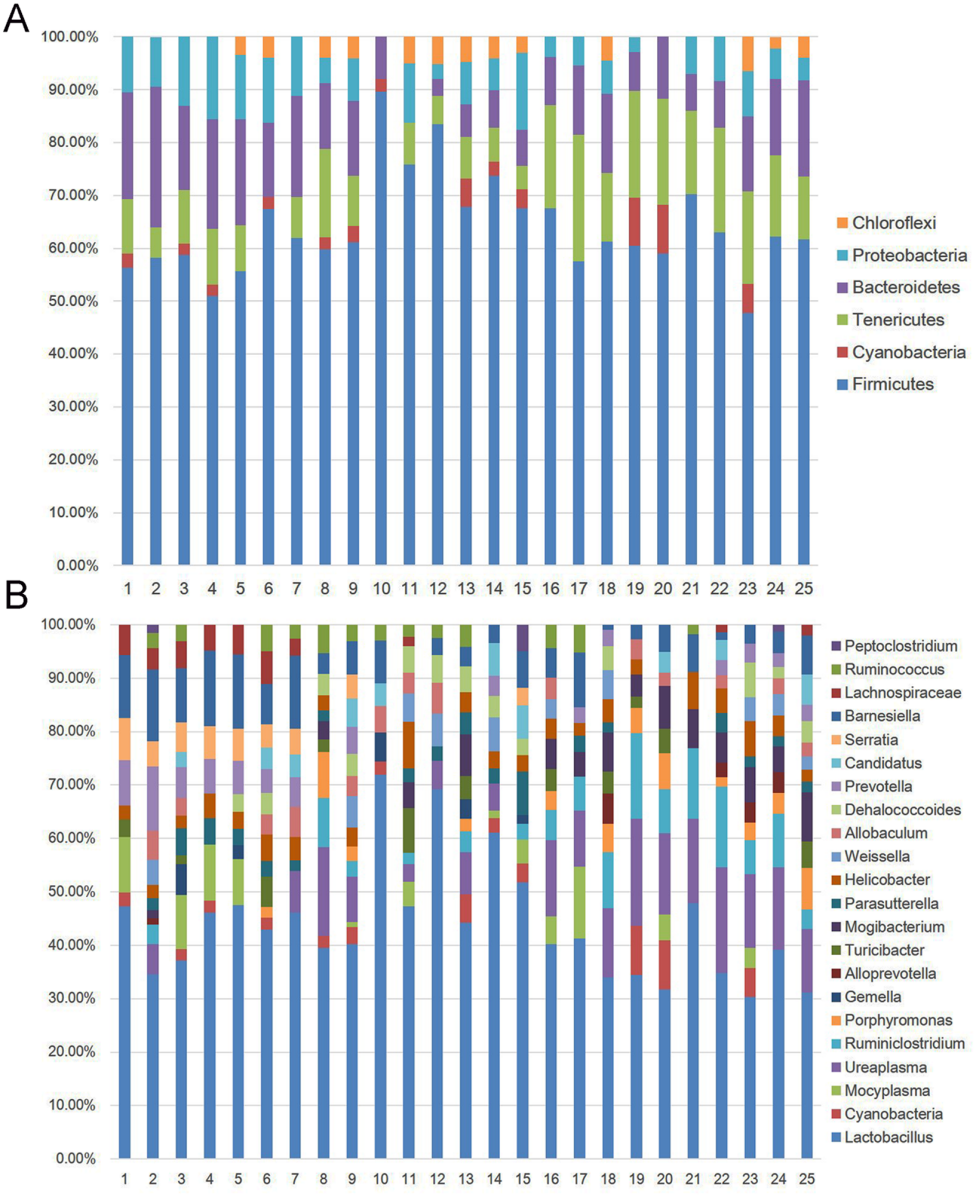
The species of microflora in duodenum, jejunum, ileum, cecum and colon of 30G, 30N, 10G, 10N and C groups without regarding to gender. (A) Phyla and (B) genus distribution of 16S rDNA clones derived from intestinal content samples. The numbers 1 to 25 were labeled as in Fig 5.

At the phylum level, we classified 16S rDNA clones into 6 phyla including *Firmicutes*, *Cyanobacteria*, *Tenericutes*, *Bacteroidetes*, *Proteobacteria* and *Chloroflexi*. *Firmicutes* presented in all the samples. *Bacteroidetes* was the prominent bacteria group except for the sample of ileum of 30G group, and *Proteobacteria* also presented in all the intestinal samples except the jejunum of C group (Fig 6A). At genus level, these clones could be classified into the following 22 genera: *Lactobacillus*, *Peptoclostridium*, *Ruminococcus*, *Lachnospiraceae*, *Barnesiella*, *Serratia*, *Candidatus*, *Prevotella*, *Dehalococcoides*, *Allobaculum*, *Weissella*, *Helicobacter*, *Parasutterella*, *Mogibacterium*, *Turicibacter*, *Alloprevotella*, *Gemella*, *Porphyromonas*, *Ruminiclostridium*, *Ureaplasma*, *Mocyplasma*, and *Cyanobacteria* (Fig 6B). *Lactobacillus* was the prominent bacteria group in all the intestinal content samples. *Barnesiella* was the preponderant bacteria group and existed at all samples except for the sample from ileum of 30G group. In duodenum, the prominent bacteria groups of all samples were similar (i.e., *Lactobacillus*, *Barnesiella* and *Mocyplasma*) except for the sample from 30N group, in which *Lactobacillus*, *Barnesiella* and *Prevotella* were the prominent bacteria groups in duodenum. In cecum and colon, each group had high similarity of prominent bacteria that were *Lactobacillus*, *Ureaplasma*, and *Ruminiclostridium*. However, the prominent bacteria groups in jejunum and ileum of each experimental group were quite variable.

**Fig 7:**
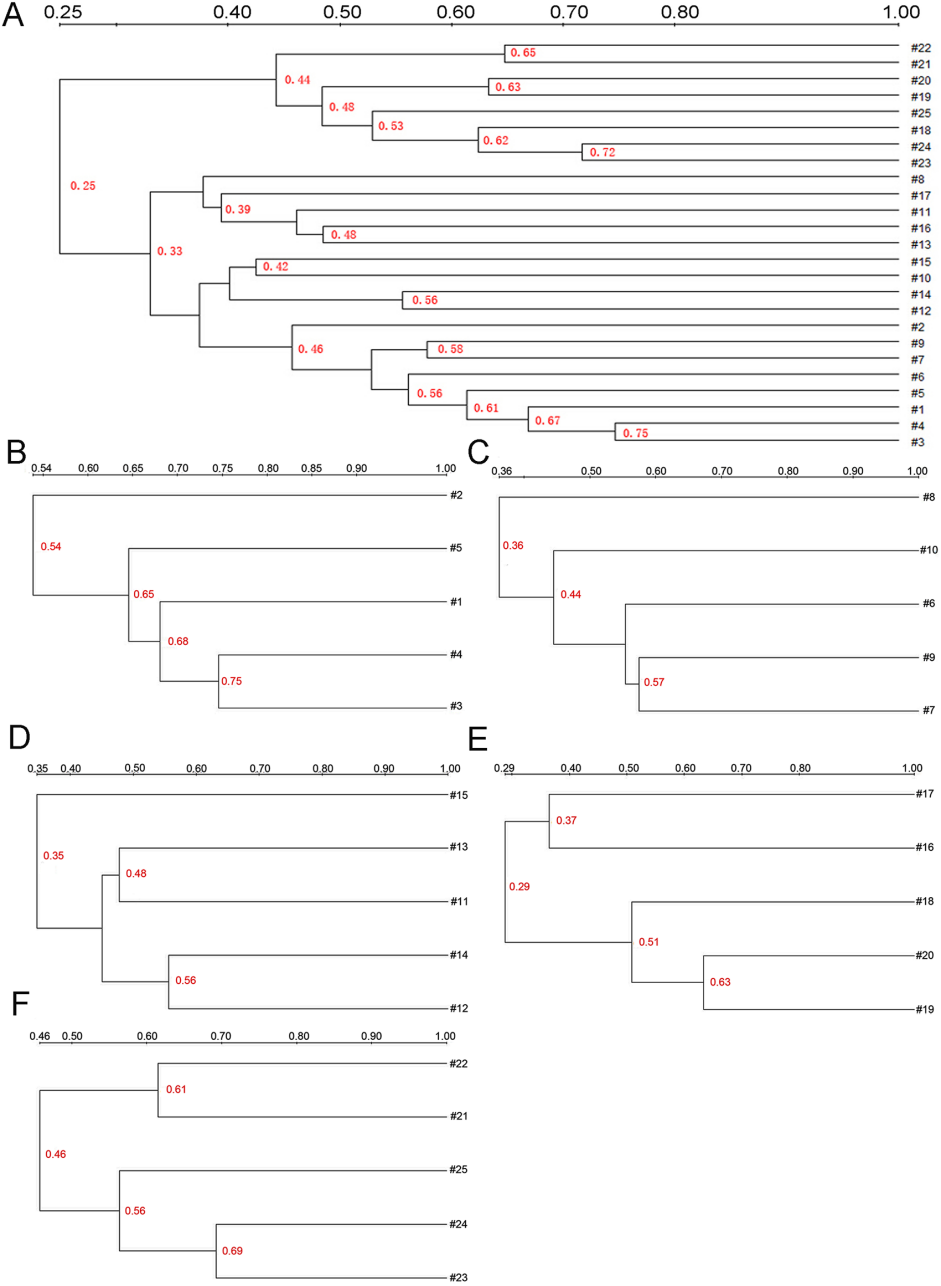
Cluster analysis of 16S rDNA clones by UPMGA. Numbers along the top indicated the similarity coefficient. The similarity coefficient of the adjacent two samples was labeled in red. The numbers 1 to 25 were labeled as those in Fig 5. A represented the cluster analysis of 16S rDNA clones of all samples, and B-F represented cluster analysis of 16S rDNA clones derived from duodenum, jejunum, ileum, cecum, and colon, respectively.

Cluster analysis showed that the similarity of all samples was 25% ~ 75% (Fig 7A). The similarity of samples in duodenum, jejunum, ileum, cecum and colon were 54% ~ 75% (Fig 7B), 36% ~ 57% (Fig 7C), 35% ~ 56% (Fig 7D), 29% ~ 63% (Fig 7E), 46% ~ 69% (Fig 7F), respectively. When the microflora in duodenum, jejunum, ileum, cecum, and colon of 30G were compared to 30N group, we found that the similarity of the two groups dropped from duodenum to cecum but rose in colon with the extension of the intestine. The similar situation was also found between 30G and C groups. The similarity of microflora between 10G and 10N groups reduced from duodenum to jejunum and rose from jejunum to colon.

**Fig 8:**
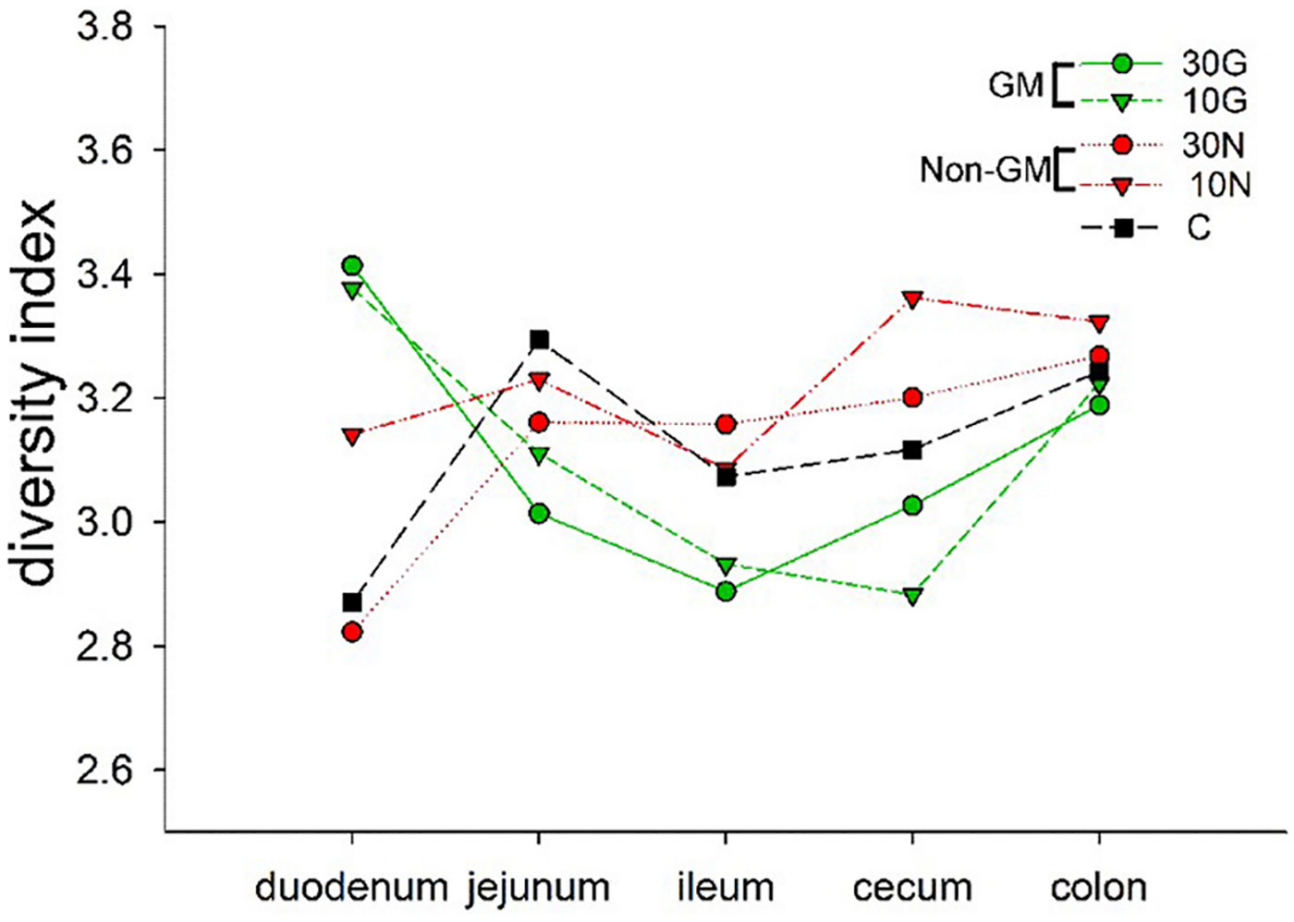
Shannon diversity analysis of intestinal microflora diversity in 30G, 30N, 10G, 10N and C groups.

In addition, microflora diversity was analyzed by Shannon diversity index. The results showed that the Shannon diversity index of duodenum was erratic in all groups, and the indices of jejunum, ileum, and cecum in GM group were lower when compared to the same segment in non-GM group or control group. However, the Shannon diversity index of colon in all groups was approximately comparable (Fig 8).

### Horizontal gene transfer was not observed

**Fig 9:**
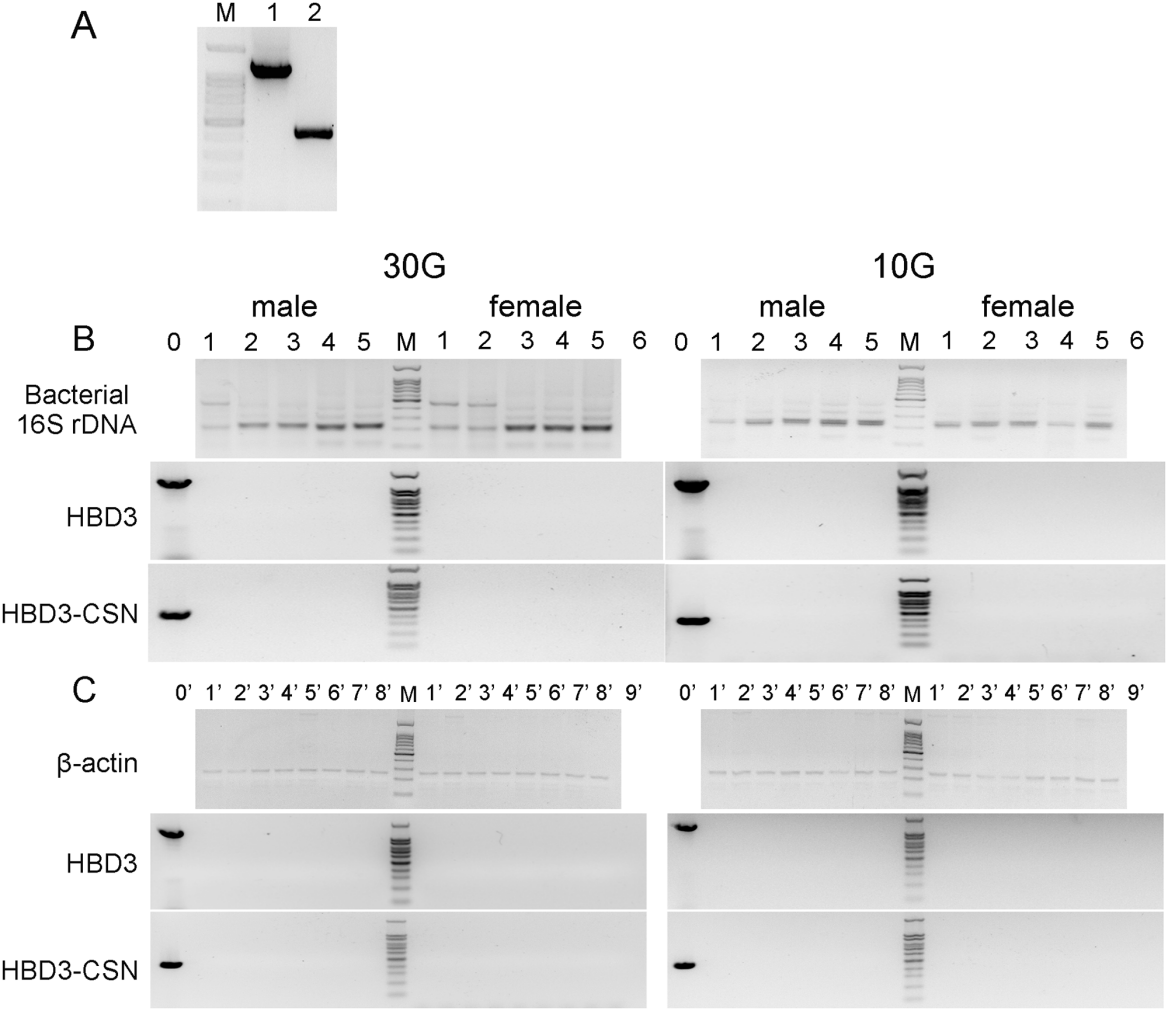
Intestinal contents and main organs were used for HGT detection by PCR. (A) DNA of transgenic cattle was used as template in PCR. Lane 1 represented the PCR was used HBD3 primers, and amplicon size was 1156bp. Lane 2 represented the PCR was used HBD3-CSN primers, and amplicon size was 415bp. M: 100 bp DNA marker. (B) HGT was detected by PCR with DNA of intestinal contents as template. 0–6 represented transgenic cattle, duodenum, jejunum, ileum, cecum, colon, and ddH_2_O, respectively. Bacterial 16S rDNA was amplified with bacteria universal primers (GC-338F and 518R), and amplicon size was 200bp. (C) HGT was detected by PCR with DNA of tissue of main organs as template. 0′-9′represented transgenic cattle, heart, liver, spleen, lung, kidney, large intestine, small intestine, muscle, and ddH_2_O, respectively. β-actin was amplified with β-actin primers, and amplicon size was 241bp. HBD3 was amplified with HBD3 primers. No product at 1156bp position was found in both 30G and 10G of intestinal contents and main organs. HBD3-CSN was amplified with HBD3-CSN primers. No product at 415bp position was found in both 30G and 10G of intestinal contents and main organs.

Previous study reported that chloroplast DNA sequences were found in chickens tissues and cows lymphocytes when the consumers were fed with GM products derived from transgenic plant, which raised the concern that exogenous gene may transfer into intestinal microflora or other tissues of the consumers [10]. To demonstrate whether HBD3 gene would transfer into the microflora or other tissues of mice after fed with diet containing HBD3, the possibility of HGT was evaluated. DNA of intestinal bacteria and tissues of heart, liver, spleen, lung, kidney, muscle, and intestine from GM groups were detected. The results showed that neither HBD3 gene nor vector fragment was found in the tested samples, indicating that there was little possibility for HGT (Fig 9).

## Discussion

Although there is no evidence that the GM animals or products have harmful effects on human or animal health, the safety assessment of GM product has been discussed for many years. And it is necessary to assess the safety of GM product in detail before it is allowed to enter into human food chain [33]. The current study was performed to determine whether adverse effects occured in GI health of mice fed with a 90-day dietary formulated with GM milk containing HBD3, and there was no significant difference in most examined variables between mice consuming diets formulated with GM milk containing HBD3 and those fed with diet produced by conventional milk or AIN93G diet.

Milk offers a large amount of essential amino acids and other nutrition, which makes it an important dietary source for human. Whether the exogenous protein of HBD3 in GM milk changed the digestibility of milk necessitates the analysis of the stability of GM milk. Here, the stability of the additional component of HBD3 protein in GM milk was measured by the simulated gastric fluid assay *in vitro*. The result of this method is influenced by the assay conditions used, especially the ratio of pepsin to test protein. In this study, the ratio of pepsin to GM milk (1:100, w/w) was much lower than the ratio used by previous studies [15, 24]. We found that HBD3 protein in milk, as well as most of milk proteins, was rapidly and completely digested within 1 min, while β-lactoglobulin remained even after 120 min digestion (around 18KD molecular weight), which is consistent with the digestion condition of conventional milk in the previous report [34].

For general health condition, we found GM milk has no adverse effects on relative organ weight of examined organs with an exception that the relative weight of spleen of male mice of 30G group was higher than control group, but no macroscopic and histopathological changes were observed in spleen. In addition, previous publications also suggested that the differences of relative organ weight of only a certain organ in the whole body between experimental and control group did not support a pathological change in GM products feeding studies [16, 17, 35]. We also should note that body weight of high concentration diet groups (30G and 30N groups) was higher than the control group, which might be largely due to extra nutrition that assimilated from high concentration diet and did not indicate that the GM milk was unsafe because the same result was observed in conventional milk group. As in the result of serum biochemistry examination, the TG level of male (30G groups) and female (30G and 30N groups) mice were higher than control group, which was supported to the ingestion of high fat and carbohydrate from high concentration diet [36] because increased TG level was also observed in conventional milk group.

Evidence from basic science and clinical studies indicate that the intestinal TJ barrier which regulate the intestinal epithelial permeability, has a critical role in the pathogenesis of intestinal and systemic diseases [37], in which TJ structure is broken, and the distribution of TJ proteins in the epithelium is abnormal. It has been reported that many factors can affect the TJ structure, such as cytokines, growth factors, pathogens, nutrients and food factors [31]. However, there is little attention of intestinal epithelial permeability which could be possibly influenced by GM products. In this study, we systematically detected the intestinal permeability at multiple aspects and levels. All the results showed that GM milk did not affect on intestinal permeability.

The microflora, which resides in GI tract, plays important roles in fermenting non-digestible residue, controlling intestinal epithelial cell proliferation and differentiation, regulating immune system development and homoeostasis [38]. In addition, the microflora serves as a physiological barrier which protects body against invasion of pathogens. The intestinal dysbacteriosis could cause a series of diseases in and out the gut [32, 38]. The microflora varies along the whole intestinal tract, and the prominent microflora in each intestinal segment could be influenced by lots of factors, such as nutrient supplies, intestinal motility, and pH [39]. In the present study, we found that the microflora diversity indicated by Shannon index analysis of jejunum, ileum, and cecum in GM groups were lower than corresponding segment of intestines in non-GM group and control group, but the microflora diversity of colon was almost identical in all the groups. This result was consistent with the study of bacteria community in gastrointestinal tract of C57BL/6 mice [39]. And the reason for this result might be that the diet was not completely digested in the upper GI tract and a small amount of undigested HBD3 protein remained in intestine. It is well known that HBD3 is a defensin with broad antimicrobial activities [40], so it could influence the intestinal microflora. The colon is far from the stomach, which offers a relatively gentle environment with less influence of diet and better for bacteria to grow [39]. The possible reason why the microflora diversity index of duodenums in each group was erratic indicated by Shannon index analysis might be that the environment of duodenums received huge impacts from adjacent stomach in which the environment is harsh because of low pH and entrance of ingested bacteria from the outer environment. Although we cannot clarify whether GM milk could affect the full GI tract, at least it does not change microflora diversity of colon which is for away from the stomach, and offers better surroundings for bacteria to grow. In addition, GM groups had similar prominent bacteria with non-GM groups and control group, suggesting that GM milk did not affect dominated bacteria in intestines.

In summary, based on the traditional subchronic toxicity study we examined the general health condition of the mice. And in addition, we put the emphasis on the effect of GM milk on the GI health including gastric emptying ability, intestinal permeability, intestinal microflora, and the possibility of HGT. The investigation about the effects of GM product on GI health could provide more evidences for the safety assessment of GM milk containing HBD3 since the digestive tract is the first site to contact food. The preliminary exploration in this study may provide detailed information about the effects of GM materials on the GI health of animals, even human being, which would consume GM products.

## Supporting Information

S1 Fig Histopathological results of duodenum, jejunum, ileum, cecum and colon of 30G, 30N, 10G, 10N, C groups. (A) Male mice and (B) female mice. Scale bar = 50 μm.
(TIF)

S2 Fig Electron microscopic analysis of the tight junctions in duodenum, jejunum, ileum, cecum, and colon of 30G, 30N, 10G, 10N, C groups. (A) Male mice and (B) female mice. TJ indicated the location of the tight junctions. AJ indicated the location of adherens junctions. DS indicated desmosomes. Scale bar = 400 nm.
(TIF)

S3 Fig mRNA relative expression of tight junction proteins zo-1, occludin, and claudin-1 in duodenum, jejunum, ileum, cecum, and colon of 30G, 30N, 10G, 10N, and C groups of male and female mice. Values are means ± SD, n = 5.
(TIF)

S4 Fig Result of immunofluorescence of zo-1, occludin, and claudin-1 in ileum and colon of male and female mice. Frozen sections of ileum and colon were labelled for zo-1 (red), occludin (red), claudin-1 (red) and nuclei (blue). Scale bar = 100 μm.
(TIF)

S5 Fig Tracer experiment to examine the permeability in ileum and colon of male and female mice. The green fluorescence signals of biotin were found to be restricted to the lumen of the ileum and colon in each group. Scale bar = 50 μm.
(TIF)

S1 Table Organ/body weight in male and female mice following 90 days. (Mean values ± SD, n = 5).
(DOCX)

S2 Table Blood biochemistry of male mice following 90 days. (Mean values ± SD, n = 5).
(DOCX)

S3 Table Blood biochemistry of female mice following 90 days. (Mean values ± SD, n = 5).
(DOCX)

S4 Table Primers of zo-1, occludin, claudin-1, and GAPDH. (DOCX)

S5 Table PCR procedure and primers used in horizontal gene transfer detection. (DOCX)

S6 Table PCR reaction volumes of horizontal gene transfer detection. (DOCX)
