# Supplementary figures and images for: Effects of Genetically Modified Milk Containing Human Beta-Defensin-3 on Gastrointestinal Health of Mice
Source: PLoS One. 2016 Jul 20;11(7):e0159700. doi: 10.1371/journal.pone.0159700 (PMC4954683; doi:10.1371/journal.pone.0159700)

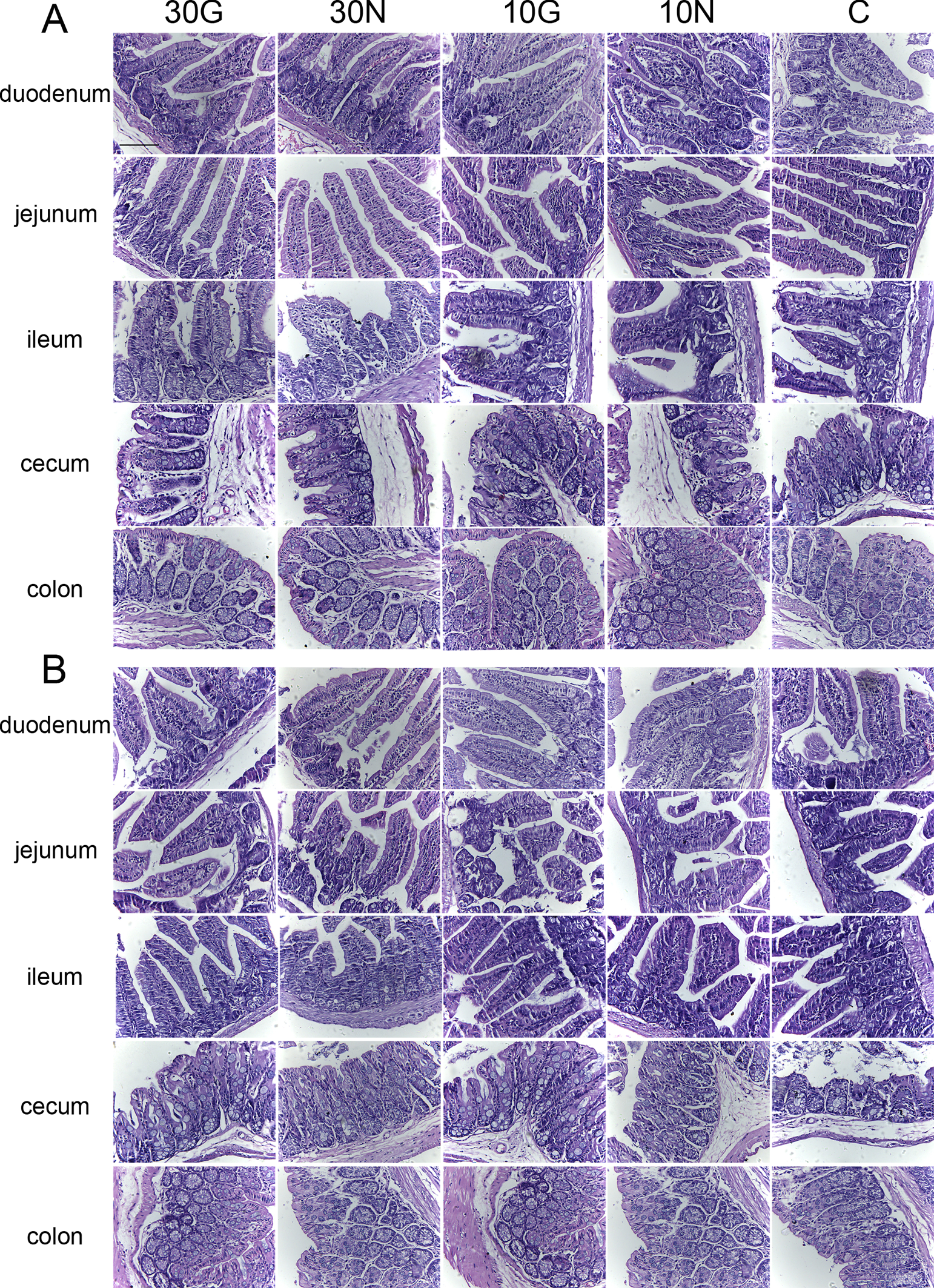

Supplement: S1 Fig — (A) Male mice and (B) female mice. Scale bar = 50 μm. (TIF) [file pone.0159700.s001.tif]

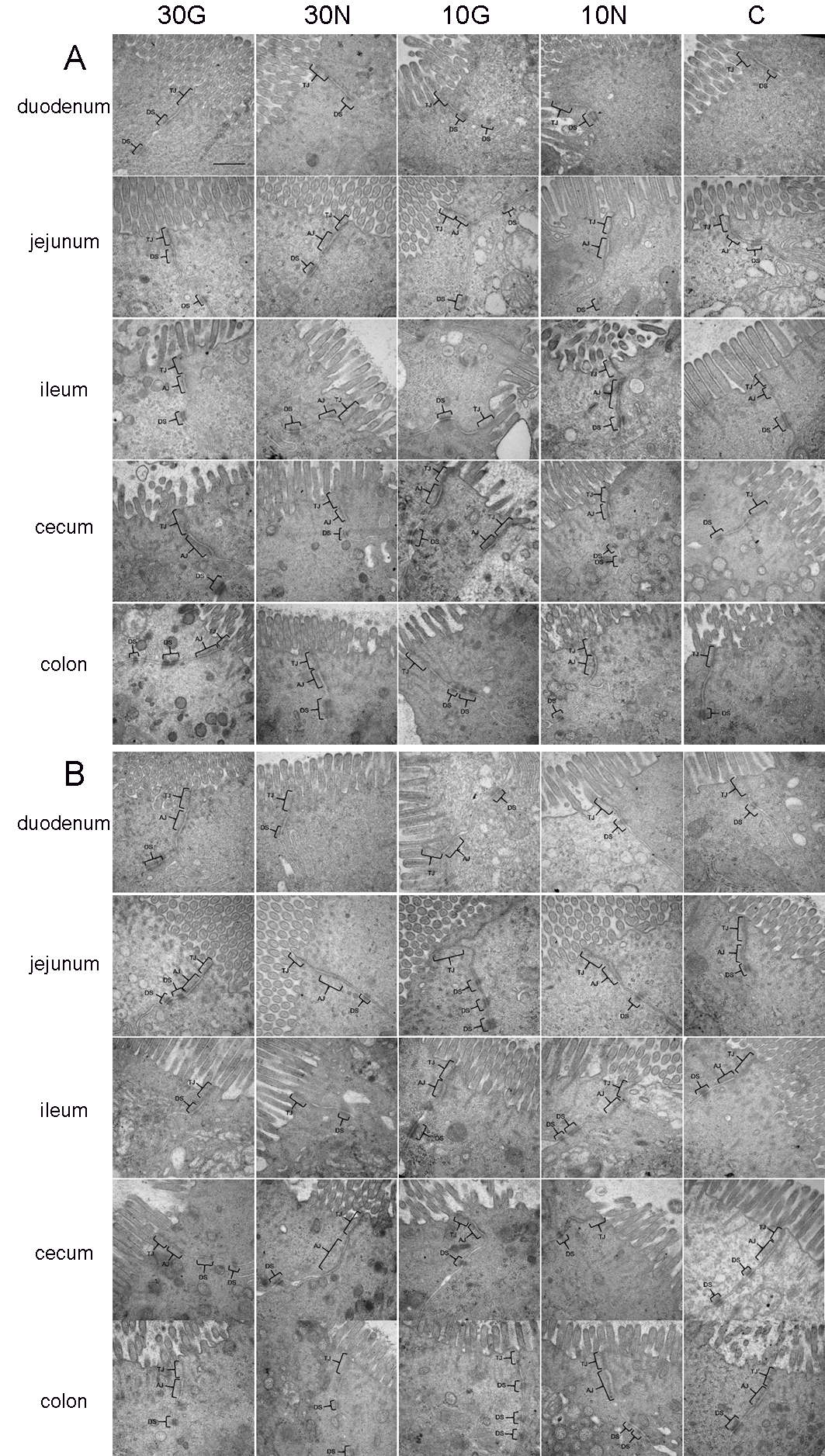

Supplement: S2 Fig — (A) Male mice and (B) female mice. TJ indicated the location of the tight junctions. AJ indicated the location of adherens junctions. DS indicated desmosomes. Scale bar = 400 nm. (TIF) [file pone.0159700.s002.tif]

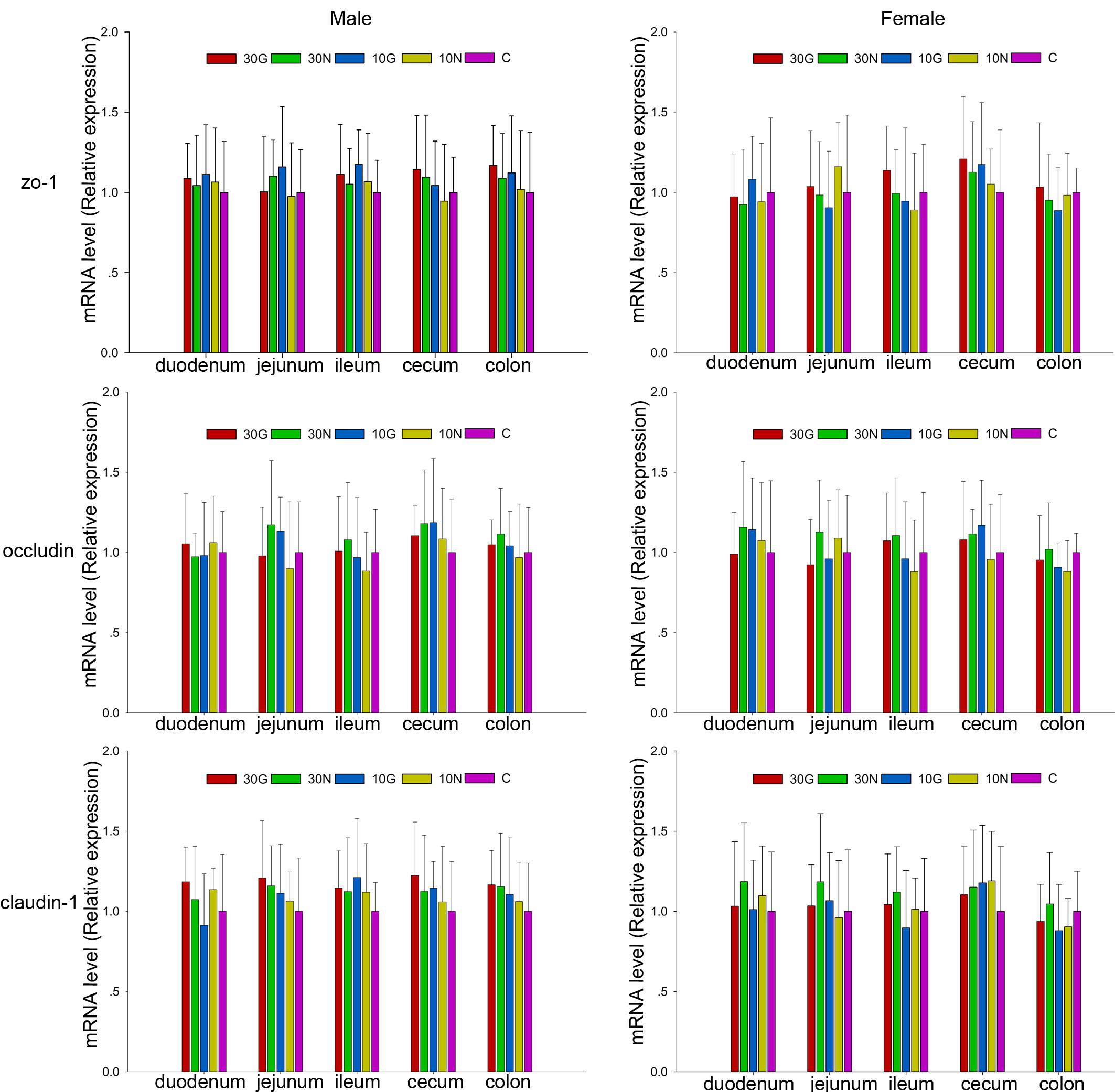

Supplement: S3 Fig — Values are means ± SD, n = 5. (TIF) [file pone.0159700.s003.tif]

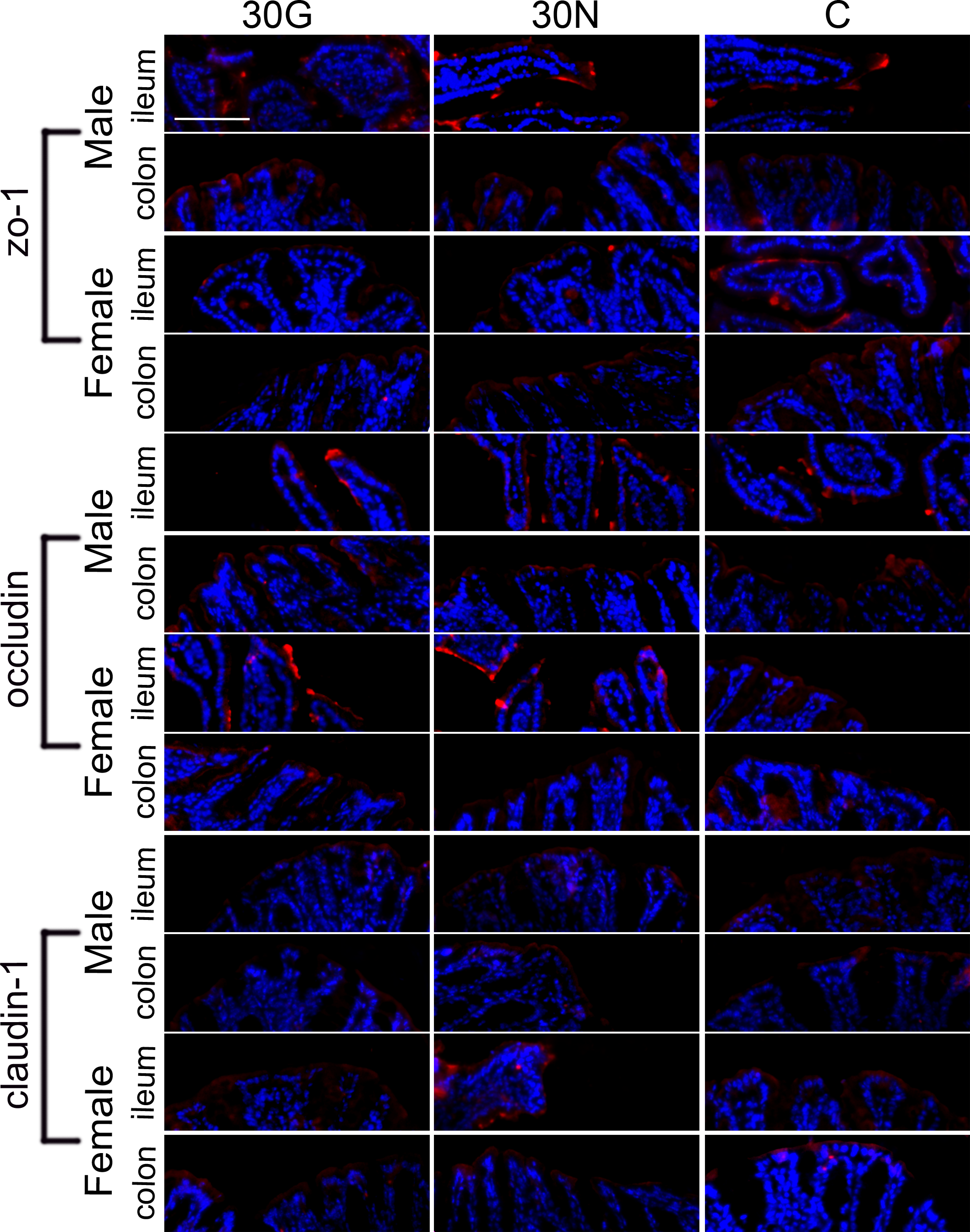

Supplement: S4 Fig — Frozen sections of ileum and colon were labelled for zo-1 (red), occludin (red), claudin-1 (red) and nuclei (blue). Scale bar = 100 μm. (TIF) [file pone.0159700.s004.tif]

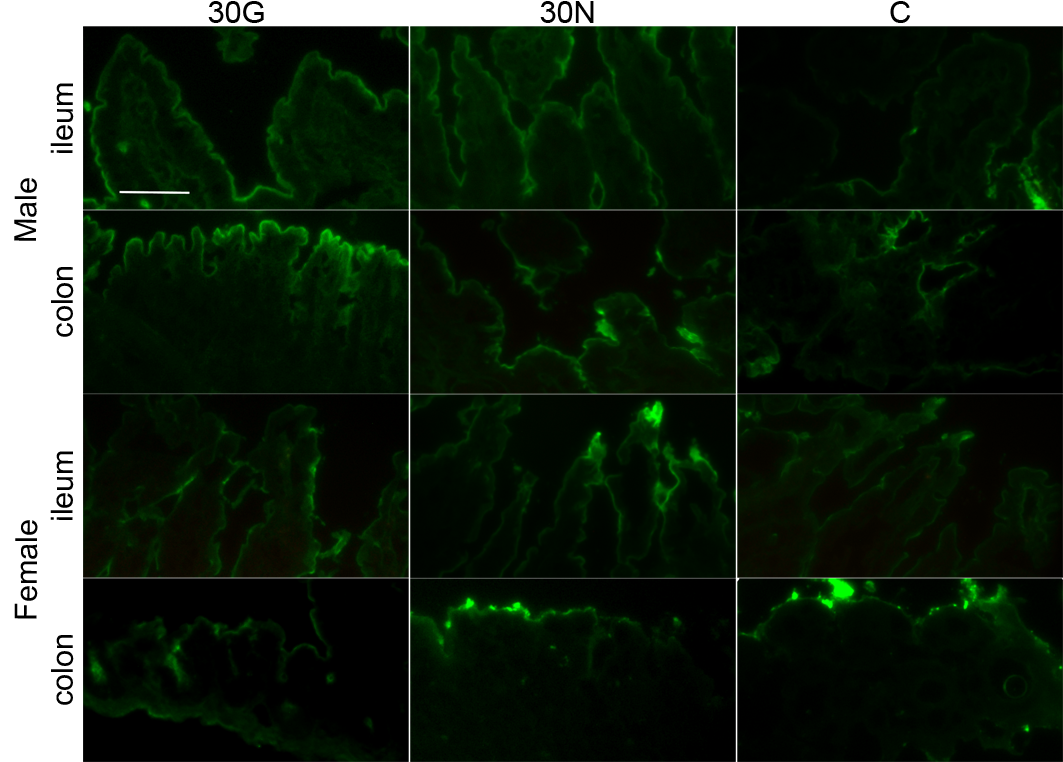

Supplement: S5 Fig — The green fluorescence signals of biotin were found to be restricted to the lumen of the ileum and colon in each group. Scale bar = 50 μm. (TIF) [file pone.0159700.s005.tif]
