# Supplementary material for: Effects of Genetically Modified Milk Containing Human Beta-Defensin-3 on Gastrointestinal Health of Mice
Source: PLoS One. 2016 Jul 20;11(7):e0159700. doi: 10.1371/journal.pone.0159700 (PMC4954683; doi:10.1371/journal.pone.0159700)
Supplement: S1 Table — (Mean values ± SD, n = 5). (DOCX) [file pone.0159700.s006.docx]

**Table S1. Organ/body weight in male and female mice following 90 days. (Mean values ± SD, n=5)**

|  | 30G | 30N | 10G | 10N | C |
| --- | --- | --- | --- | --- | --- |
| **Male** |  |  |  |  |  |
| heart | 0.762±0.10 | 0.686±0.15 | 0.616±0.14 | 0.559±0.01 | 0.626±0.10 |
| Liver | 4.524±0.54 | 5.065±0.46 | 4.719±0.17 | 4.486±0.47 | 4.951±0.48 |
| spleen | 0.661±0.21 * | 0.459±0.07 | 0.379±0.19 | 0.342±0.02 | 0.412±0.16 |
| lungs | 0.858±0.25 | 0.682±0.11 | 0.673±0.11 | 0.710±0.08 | 0.730±0.16 |
| kidneys (paired) | 1.541±0.29 | 1.290±0.13 | 1.172±0.15 | 1.352±0.09 | 1.285±0.17 |
|  |  |  |  |  |  |
| **Female** |  |  |  |  |  |
| heart | 0.685±0.39 | 0.544±0.06 | 0.640±0.09 | 0.576±0.12 | 0.501±0.04 |
| liver | 4.742±0.89 | 4.70±0.63 | 4.680±0.62 | 4.279±0.49 | 4.659±0.33 |
| spleen | 0.475±0.14 | 0.397±0.11 | 0.445±0.13 | 0.462±0.14 | 0.404±0.10 |
| lungs | 2.182±1.42 | 1.359±0.73 | 2.103±1.20 | 1.334±0.64 | 1.611±0.77 |
| kidneys (paired) | 1.221±0.08 | 1.138±0.17 | 1.203±0.10 | 1.220±0.22 | 1.162±0.10 |

* p < 0.05 versus C group
