# Supplementary material for: Effects of Genetically Modified Milk Containing Human Beta-Defensin-3 on Gastrointestinal Health of Mice
Source: PLoS One. 2016 Jul 20;11(7):e0159700. doi: 10.1371/journal.pone.0159700 (PMC4954683; doi:10.1371/journal.pone.0159700)
Supplement: S2 Table — (Mean values ± SD, n = 5). (DOCX) [file pone.0159700.s007.docx]

**Table S2. Blood biochemistry of male mice following 90 days. (Mean values ± SD, n=5)**

| Blood biochemistry | **30G** | **30N** | **10G** | **10N** | **C** |
| --- | --- | --- | --- | --- | --- |
| ALT（U/L） | 39.13±2.43 | 38.80±1.82 | 39.30±2.41 | 40.03±3.13 | 38.50±4.06 |
| AST（U/L） | 173.20±30.75 | 167.88±11.71 | 175.10±25.50 | 168.05±20.82 | 166.4±55.70 |
| ALP（U/L） | 130.88±23.64 | 101.95±12.46 | 105.50±12.35 | 103.68±7.99 | 108.67±6.43 |
| TP（g/L） | 52.20±2.07 | 52.3±1.62 | 52.00±1.62 | 52.03±1.08 | 52.67±1.76 |
| ALB（g/L） | 17.30±0.34 | 17.23±0.35 | 17.20±0.45 | 17.23±0.39 | 17.30±0.26 |
| GLB（g/L） | 34.90±2.36 | 35.08±1.51 | 34.80±1.18 | 34.80±0.80 | 35.37±1.63 |
| A/G | 0.50±0.04 | 0.49±0.02 | 0.49±0.01 | 0.49±0.01 | 0.49±0.02 |
| TBIL（mmol/L） | 1.00±0.14 | 0.90±0.08 | 0.90±0.16 | 0.93±0.13 | 0.93±0.06 |
| TG（mmol/L） | 1.17±0.32*^△^ | 0.72±0.11 | 0.74±0.20 | 0.75±0.12 | 0.72±0.17 |
| T-CHOL（μmol/L） | 1.44±0.10 | 1.44±0.13 | 1.47±0.18 | 1.43±0.15 | 1.44±0.16 |
| TBA（μmol/L） | 1.63±0.26 | 1.68±0.50 | 1.65±0.37 | 1.65±0.37 | 1.67±0.90 |
| CHE（U/L） | 3792.25±278.81 | 3830.25±273.22 | 3841.25±316.30 | 3826.00±160.50 | 3808.67±306.27 |

* p < 0.05 versus C group，^△^p < 0.05 versus 30N group.

ALT: alanine aminotransferase, AST: aspartate aminotransferase, ALP: alkaline phosphatase, TP: total protein, ALB: albumin, GLO: globulin, TBIL: total bilirubin, TBA: total bile acid, CHE: cholinesterase, T-CHOL: total cholesterol, and TG: triglyceride
