# Supplementary material for: Effects of Genetically Modified Milk Containing Human Beta-Defensin-3 on Gastrointestinal Health of Mice
Source: PLoS One. 2016 Jul 20;11(7):e0159700. doi: 10.1371/journal.pone.0159700 (PMC4954683; doi:10.1371/journal.pone.0159700)
Supplement: S3 Table — (Mean values ± SD, n = 5). (DOCX) [file pone.0159700.s008.docx]

**Table S3. Blood biochemistry of female mice following 90 days. (Mean values ± SD, n=5)**

| Blood biochemistry | **30G** | **30N** | **10G** | **10N** | **C** |
| --- | --- | --- | --- | --- | --- |
| ALT（U/L） | 37.78±1.95 | 36.08±0.64 | 36.43±0.69 | 36.23±1.18 | 36.37±0.81 |
| AST（U/L） | 196.68±34.25 | 205.78±30.11 | 215.50±20.94 | 209.35±32.32 | 212.57±36.89 |
| ALP（U/L） | 89.35±25.97 | 83.55±11.22 | 81.80±10.36 | 86.93±10.62 | 79.25±23.68 |
| TP（g/L） | 60.63±2.87 | 61.58±1.65 | 61.20±1.58 | 61.43±1.97 | 61.1±2.26 |
| ALB（g/L） | 19.60±0.96 | 19.68±1.37 | 19.45±0.82 | 19.33±0.72 | 19.25±1.72 |
| GLB（g/L） | 41.02±3.16 | 41.88±0.66 | 41.75±2.17 | 42.1±1.85 | 41.85±1.46 |
| A/G | 0.48±0.06 | 0.47±0.03 | 0.47±0.04 | 0.46±0.03 | 0.46±0.05 |
| TBIL（mmol/L） | 0.75±0.10 | 0.75±0.13 | 0.70±0.14 | 0.73±0.10 | 0.73±0.15 |
| TG（mmol/L） | 0.90±0.17* | 0.86±0.27* | 0.56±0.20 | 0.53±0.13 | 0.55±0.16 |
| T-CHOL（μmol/L） | 0.96±0.07 | 0.95±0.07 | 0.97±0.05 | 0.94±0.01 | 0.94±0.04 |
| TBA（μmol/L） | 4.56±0.65 | 4.78±0.48 | 4.73±0.69 | 4.68±0.46 | 4.63±0.78 |
| CHE（U/L） | 5630.00±280.96 | 5668.00±406.09 | 5707.50±471.15 | 5615.00±545.48 | 5652.25±825.12 |

* p < 0.05 versus C group
