# Supplementary material for: Effects of Genetically Modified Milk Containing Human Beta-Defensin-3 on Gastrointestinal Health of Mice
Source: PLoS One. 2016 Jul 20;11(7):e0159700. doi: 10.1371/journal.pone.0159700 (PMC4954683; doi:10.1371/journal.pone.0159700)
Supplement: S4 Table — (DOCX) [file pone.0159700.s009.docx]

**Table S4. Primers of zo-1, occludin, claudin-1, and GAPDH.**

| Target | Sequence (5’–3’) |
| --- | --- |
| zo-1 | F: 5’-GCCGCTAAGAGCACAGCAA-3’ |
|  | R: 5’-TCCCCACTCTGAAAATGAGGA-3’ |
| occludin | F: 5’-CTGGATCTATGTACGGCTCACA-3’ |
|  | R: 5’-TCCACGTAGAGACCAGTACCT-3’ |
| claudin-1 | F: 5’-GGGGACAACATCGTGACCG-3’ |
|  | R: 5’-AGGAGTCGAAGACTTTGCACT-3’ |
| GAPDH | F: 5’-GTGTTCCTACCCCCAATGTGT-3’ |
|  | R: 5’-ATTGTCATACCAGGAAATGAGCTT-3’ |
