# Supplementary material for: Effects of Genetically Modified Milk Containing Human Beta-Defensin-3 on Gastrointestinal Health of Mice
Source: PLoS One. 2016 Jul 20;11(7):e0159700. doi: 10.1371/journal.pone.0159700 (PMC4954683; doi:10.1371/journal.pone.0159700)
Supplement: S5 Table — (DOCX) [file pone.0159700.s010.docx]

**Table S5. PCR procedure and primers used in horizontal gene transfer detection.**

| Target | Annealing temperature | | Amplicon size （bp） | Sequence (5’-3’) |
| --- | --- | --- | --- | --- |
| ß-actin | | 53℃ | 241 | F: GGCTGTATTCCCCTCCATCG |
|  |  |  |  | R: CCAGTTGGTAACAATGCCATGT |
| Bacterial 16S rDNA | | 55℃ | 200 | GC-338F: CGCCCGGGGCGCGCCCCGGGGCGGGGCGGGGGCGCGGGGGGCCTACGGGAGGCAGCAG |
|  |  |  |  | 518R: ATTACCGCGGCTGCTGG |
| HBD3 | | 55℃ | 1156 | F: CCGCTCGAGAGCAGCTATGAGGATCCA |
|  |  |  |  | R: TATCGATCGTTTTATTTCTTTCTTCGG |
| HBD3-CSN | | 55℃ | 415 | F: CTTCCTAAAACCTTTCCGTG |
|  |  |  |  | R: CCAGTCGCAGTCAATTCTGT |
