# Supplementary material for: Effects of Genetically Modified Milk Containing Human Beta-Defensin-3 on Gastrointestinal Health of Mice
Source: PLoS One. 2016 Jul 20;11(7):e0159700. doi: 10.1371/journal.pone.0159700 (PMC4954683; doi:10.1371/journal.pone.0159700)
Supplement: S6 Table — (DOCX) [file pone.0159700.s011.docx]

**Table S6. PCR reaction volumes of horizontal gene transfer detection.**

| Reagent | Final concentration | Volume (μl) |
| --- | --- | --- |
| 10x TransTaq HiFi Buffer | 1x | 5 |
| 10 μmol/L Primer Mix | 0.4 μmol/L | 2 |
| 100 ng/μl DNA template | 2 ng/μl | 1 |
| 2.5 mM dNTPs | 0.2 mM | 4 |
| TransTaq HiFi DNA Polymerase | - | 1 |
| ddH2O | - | 37 |
| reaction volumes | - | 50 |
